# Development of Morphologically engineered Flower-like Hafnium-Doped ZnO with Experimental and DFT Validation for Low-Temperature and Ultrasensitive Detection of NO_X_ Gas

**DOI:** 10.1021/acs.iecr.2c00890

**Published:** 2022-04-22

**Authors:** Srijita Nundy, Sankar Ganesh Ramaraj, Manoharan Muruganathan, Aritra Ghosh, Asif Ali Tahir, Tapas Kumar Mallick, Joon-Shik Park, Hoo-Jeong Lee

**Affiliations:** † School of Advanced Materials and Science Engineering, Sungkyunkwan University, Suwon 16419, Republic of Korea; ‡ School of Materials Science, Japan Advanced Institute of Science and Technology, Nomi 923-1211, Japan; § SKKU Advanced Institute of Nano Technology, Sungkyunkwan University, Suwon 16419, Republic of Korea; ∥ Smart Sensor Research Center, Korea Electronics Technology Institute (KETI), Seongnam 13509, Republic of Korea; ⊥ College of Engineering, Mathematics and Physical Sciences, Renewable Energy, University of Exeter, Penryn TR10 9FE, United Kingdom; # Environment and Sustainability Institute, University of Exeter, Penryn TR10 9FE, United Kingdom

## Abstract

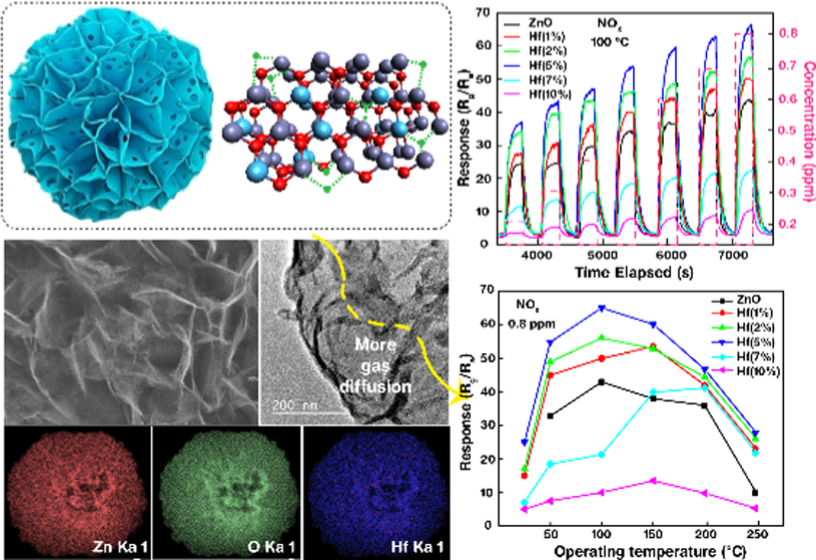

Substitutional doping and different nanostructures of ZnO have rendered it an effective sensor for the detection of volatile organic compounds in real-time atmosphere. However, the low selectivity of ZnO sensors limits their applications. Herein, hafnium (Hf)-doped ZnO (Hf-ZnO) nanostructures are developed by the hydrothermal method for high selectivity of hazardous NO_X_ gas in the atmosphere, substantially portraying the role of doping concentration on the enhancement of structural, optical, and sensing behavior. ZnO microspheres with 5% Hf doping showed excellent sensing and detected 22 parts per billion (ppb) NO_X_ gas in the atmosphere, within 24 s, which is much faster than ZnO (90 s), and rendered superior sensing ability (*S* = 67) at a low temperature (100 °C) compared to ZnO (*S* = 40). The sensor revealed exceptional stability under humid air (*S* = 55 at 70% RH), suggesting a potential of 5% Hf-ZnO as a new stable sensing material. Density functional theory (DFT) and other characterization analyses revealed that the high sensing activity of 5% Hf-ZnO is attributed to the accessibility of more adsorption sites arising due to charge distortion, increased oxygen vacancies concentration, Lewis acid base, porous morphology, small particle size (5 nm), and strong bond interaction amidst NO_2_ molecule with ZnO-Hf-O_vacancy_ sites, resulting from the substitution of the host cation (Zn^2+^) with doping cation (Hf^4+^).

## 1 Introduction

Currently, the major atmospheric pollutants are oxides of nitrogen (collectively termed NO_X_), which cause smog, acid rain, and respiratory diseases.^1−4^ To tackle this challenge, accurate detection of NO_X_ using stable and selective gas sensors is paramount. Among various gas sensing metal oxides (MOS: SnO_2_, TiO_2_, etc.), ZnO is significant because of its NO_X_ detection ability with high sensitivity and selectivity.^5−7^ However, high-temperature (over 200 °C) operation is a major weakness of MOS sensors.^8,9^ By changing the microstructure, morphology, or enhancing defects, the performance of ZnO-based sensors can also be effectively improved, which can be achieved by engaging several synthesis methods,^10^ UV illumination, or employing dopants.^11^

Doping of semiconductor materials with transition metals (TM: Al, Cr, Sn, Mn, Ni, Co, Fe, and Cu) and rare earth metals (La and Tb) has been extensively used to enhance gas sensing properties^12,13^ owing to lattice distortions in the host lattice, surface defects, especially the generation of oxygen vacancies, surface morphology variation, and grain size refinements. Transition metal hafnium (Hf) occurs in a different oxidation state (+4), resulting in the charge mismatch between Hf^4+^ and Zn^2+^ and possesses an ionic radius similar (0.78 Å) to Zn (0.74 Å).^14,15^ Also, it has a low electronegativity (1.3) and high basicity, which makes Hf a favorable candidate for doping ZnO to be used as a NO_X_ sensor application. Gas sensing activity of metal oxide nanoparticles increases as a result of oxygen vacancy enhancement. Charge transfer between ZnO NPs and doped TM ions generates oxygen vacancies, alters neighboring cations valence states, and forms a donor state within the band gap.^16^ Thus, enhanced gas responses from ZnO are possible by doping it with TM, which can have charge mismatch.^17,18^ Furthermore, NO_2_, being acidic in nature, is easily affected by Hf (basic), resulting in a substantial interaction between Lewis acid and base^19^ amidst the metal oxide and NO_X_ molecules, thereby impacting the overall superior gas sensing response.

Another point to be considered in the case of doping is the critical doping concentration of the dopant into the host lattice, where at a high doping concentration (above the critical point), hafnium aggregates on the surface of ZnO, generating surface roughness.^14,20^ Studies of the luminescence properties of Hf-ZnO have shown that hafnium induces defects (green emission) that are associated with oxygen vacancies.^20,21^ However, to our knowledge, the sensing properties of Hf-ZnO for NO_X_ detection have not been reported previously. Thus, it will be interesting to explore the effect of different hafnium doping concentrations on the properties of host metal oxides and their corresponding gas sensing behavior.

Herein, we investigated the role of hafnium doping on the morphology, microstructure, and defects modulation of the ZnO microsphere and thereby studied the effects of Hf doping on the improvement of ZnO-based NO_X_ gas sensor, in terms of selectivity, stability, and fast response toward the target NO_X_ gas. We fabricated various concentrations of Hf-doped ZnO (1, 3, 5, 7, 10% Hf) porous microsphere-based NO_X_ sensors by adapting our previously reported hydrothermal synthesis strategy^22^ for developing ZnO microspheres, which involved annealing the zinc hydroxide carbonate precursor in a vacuum environment to produce highly defective and porous microsphere for high NO_X_ sensing. The correlation between different dopant concentrations toward oxygen vacancies and the properties of the sensing materials were investigated by employing various characterization techniques (X-ray diffraction (XRD), scanning electron microscopy (SEM), transmission electron microscopy (TEM), photoluminescence (PL), X-ray photoelectron spectroscopy (XPS)) and theoretical modeling using density functional theory (DFT). We established a comparative gas sensing behavior between the pristine and doped ZnO-based gas sensors. The excellent sensing response (*S* = 67) of the 5% Hf-ZnO sensor toward a very low concentration (0.8 ppm) of NO_X_ gas at a low temperature (100 °C) was obtained, which was further investigated to understand the mechanism behind such behavior.

## 2 Material and Methods

### 2.1 Fabrication of Hf-ZnO-Based Gas Sensors

**Figure 1:**
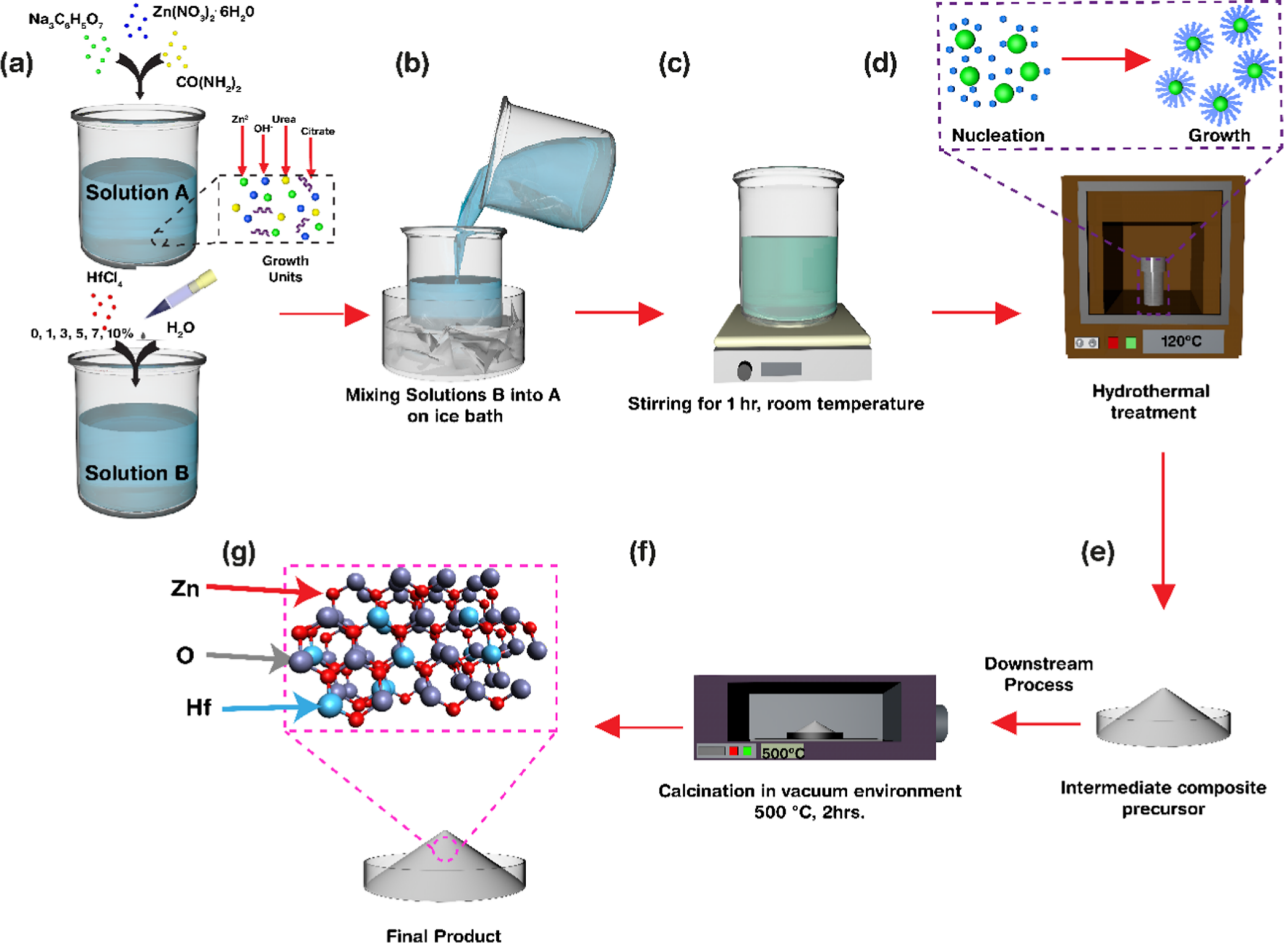
Schematic representation of the synthesis and growth process of pure and Hf-ZnO nanostructures from the precursors. (a–c) Preparation of precursor solution by mixing Zn(NO_3_)_2_·H_2_O, sodium citrate, and urea with different wt % of hafnium (1, 3, 5, 7, 10%) in DIW, (d) hydrothermal synthesis at 120 °C, 3 h along with growth mechanism of the intermediate precursor, (e) intermediate Zn-Hf precursor, and (f) calcination in a furnace under vacuum, 500 °C for 2 h. (g) Final products obtained after processing.

All of the chemicals purchased from Merck are of analytical grade and have been used as received without further purification or modification, unless mentioned otherwise. The sensing materials employed here have been prepared via our previously reported work.^22^ In brief, zinc nitrate hexahydrate (Zn(NO_3_)_2_·H_2_O, 3 mmol), urea (CO(NH_2_)_2_, 4 mmol), and trisodium citrate (Na_3_C_6_H_5_O_7_, 0.3 mmol) were dissolved in 100 mL of de-ionized water (DIW) to make a solution, which was then further prepared with different aqueous solutions of hafnium IV chloride (3–10 wt %) via constant vigorous stirring to form a transparent solution. The resultant mixture was then transferred inside a 100 mL Teflon-lined autoclave and was maintained at 120 °C for 5 h and then allowed to cool automatically to room temperature. The white precipitate collected was then taken for several washing steps with DIW and ethanol via centrifugation (5000 rpm, 15 min) followed by drying in air (80 °C for 10 h). The resultant powder was annealed in a vacuum (6 × 10^–5^ torr) furnace at 500 °C (10 °C/min) for 2 h (Figure 1). The synthesized materials with 0, 1, 3, 5, 7, and 10 wt % Hf doping are labeled as ZnO, 3% Hf-ZnO, 5% Hf-ZnO, 7% Hf-ZnO, and 10% Hf-ZnO, respectively, with other experimental parameters remaining constants. The pristine ZnO was prepared with the above-mentioned process without addition of any hafnium content.

### 2.2 Material Characterization

The detailed microstructural analyses of the as-synthesized sensing materials were executed by X-ray powder diffraction (XRD phase analysis using Bruker D8 Advance diffractometer with Cu Kα radiation), field emission scanning electron microscopy (FE-SEM morphology analysis using JEOL, JSM-7600F), high-resolution transmission electron microscopy (HR-TEM microstructural analysis using JEOL, JEM-2100F, 200 kV), Brunauer–Emmett–Teller and Barrett–Joyner–Halenda (BET–BJH surface and pore analysis using BEL, Belsorp-Mini II) analysis, photoluminescence spectroscopy (PL, defect analysis using a Scinco fluorimeter FS-2, Xe excitation at 350 nm), and X-ray photoelectron spectroscopy (XPS, for chemical state and defects analysis using a Thermo Scientific, ESCALAB 250Xi spectrometer, 1450 eV monochromatic Al Kα X-ray, 650 μm spot size). All of the density functional theory (DFT) simulations were carried out using the Quantum ATK DFT package,^23,24^ which is based on a linear combination of numerical atomic orbitals. FHI pseudopotentials with double ζ polarized (DZP) basis set were employed. To accurately account for the long-range van der Waals (vdW) interaction of ZnO and gas molecules, Grimme DFT-D2 van der Waals corrections were utilized.^25^ The Perdew–Burke–Ernzerhof (revPBE) exchange–correlation functional was employed, and a 15 Å vacuum distance was used above the ZnO layer to overcome any spurious interactions with the adjacent supercell. A density mesh cutoff of 100 Ha was employed in these simulations.

### 2.3 Sensor Fabrication and Gas Response

Interdigitated electrode-based sensor devices were fabricated by sputtering 10 nm of titanium and 150 nm of platinum electrodes (10 μm electrode distance) on a silicon oxide thin-film (300 nm)-coated substrate, followed by photolithography and dry etching process as reported in our previous work.^22^ The as-synthesized sensing materials (0.5 wt % dispersed in DIW) were drop-casted on the substrate accompanied by heating on a hot plate (100 °C), to evaporate the solvent. The devices were further taken for annealing in a muffle furnace (350 °C, 2 h) before exposing to a gas chamber. In-house gas sensing experiments were conducted with oxides of nitrogen, toluene, acetone, and ammonia, which were diluted with 2 L/min (2000 sccm) of synthetic air adjusted using mass flow controllers (MFCs). The target gas dilution calculations are shown in eq 1
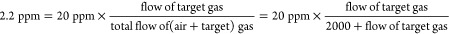
1where flow of target gas is 275 sccm.

The temperature of the gas chamber was varied from room temperature to 300 °C, employing a Joule heating system using a ceramic heater adjoined to the power supply. The adjacent electrical resistances and gas sensing responses were obtained and evaluated using a digital multimeter and data acquisition software (FLUKE). The sensor responses were calculated as  (oxidizing gas) and  (reducing gas), where *R*_a_ and *R*_g_ are the resistances of the sensor in air and in the presence of target gas, respectively. The gas sensing measurements were further evaluated by introducing humid air (combination of dry synthetic air with 100% humid air cylinder controlled by MFCs). The final humidity within the chamber was monitored using a humidity sensor (Farnell, T9602-3-D, U.K.).

## 3 Results and Discussion

### 3.1 Pure and Doped Hf-ZnO Porous Microstructures: Structural Characterization

**Figure 2:**
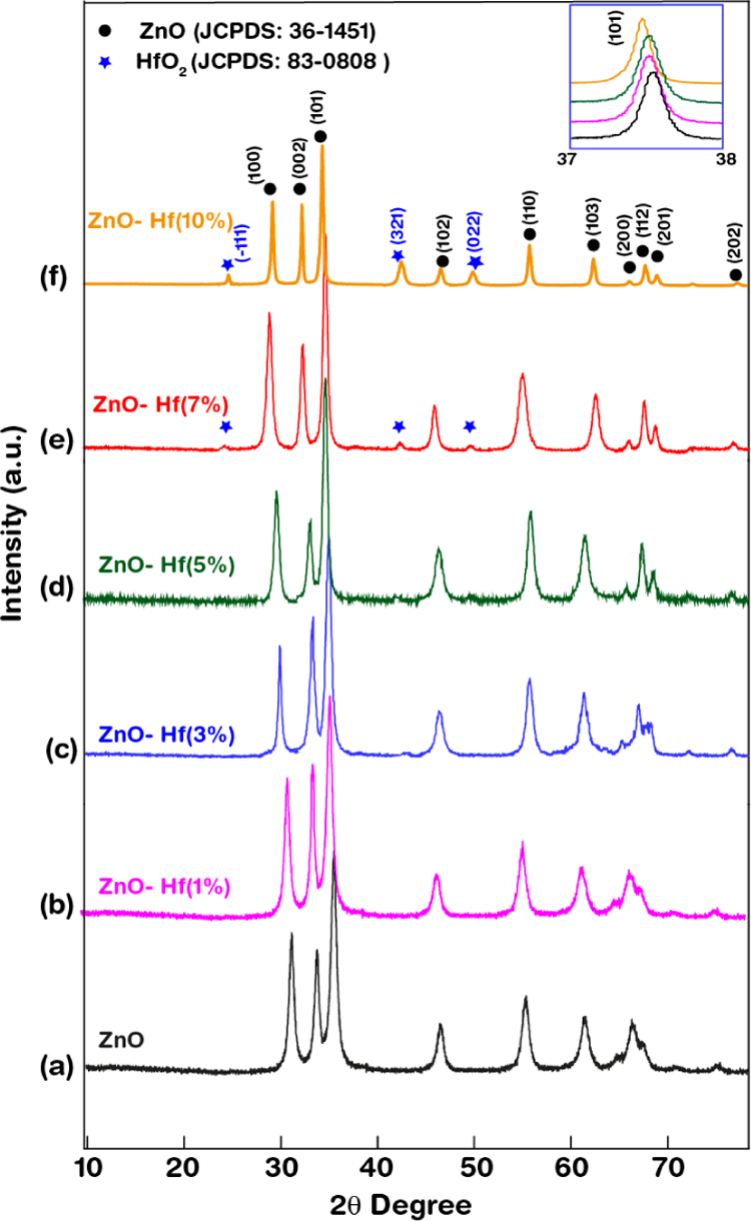
XRD spectra of pure and Hf-ZnO samples. Inset: High-resolution image of (101) peak shift toward the lower angle with an increase in doping concentration.

XRD spectra of the as-synthesized pure and Hf-ZnO microstructures are shown in Figure 2. All of the diffraction peaks were indexed well with the crystalline hexagonal wurtzite phase of ZnO (JCPDS No. 36-1451).^26^ No peaks corresponding to HfO_2_ or mixed oxides were evident in the materials prepared with hafnium doping concentration ≤5%. However, weak intensity peaks corresponding to a HfO_2_ phase started to appear in the materials containing a doping concentration of 7% at 2θ = 28.3° corresponding to the (1̅11) plane of cubic HfO_2_ (JCPDS No. 83-0808).^27^ As the doping concentration increased, the intensity of the peaks at 42 and 51.8° emerged, which are related to the (321) and (022) planes of HfO_2_.^27^ This implied that 5% was the critical doping point at which the dopant ions (Hf^4+^) were well incorporated into the ZnO lattice by substituting the host ions (Zn^2+^), whereas a secondary phase of HfO_2_ starts forming at higher doping concentrations. Further, this small shift toward a lower 2θ was observed with increasing doping concentration, indicating lattice distortion, owing to the successful substitution of Zn with Hf, thereby causing a decrease in the lattice parameters. Additionally, a minor peak widening was also observed with an increase in doping concentration until 5% Hf-ZnO, suggesting a reduction in the crystallite size. The crystallite size was further estimated using the Scherrer method, as shown in Table 1. The average crystallite size decreased from 10.92 to 8.11 nm until 5% doping and increased to 19.84 and 30.26 nm after further doping with 7 and 10% Hf, respectively.

**Table 1:** Crystallite Size Determination of Pure and Hf-ZnO Samples Using FWHM and the Scherrer Equation

sample	peak position (2θ) (101)	FWHM (width)	XRD: crystallite size *D* (nm)	TEM: particle size *D* (nm)
ZnO	36.19	0.765	10.92	9.5
1% Hf-ZnO	36.12	0.868	9.63	7.5
3% Hf-ZnO	36.09	0.963	8.68	7.25
5% Hf-ZnO	36.04	1.030	8.11	5.0
7% Hf-ZnO	36.01	0.421	19.84	52.5
10% Hf-ZnO	35.94	0.276	30.26	

**Figure 3:**
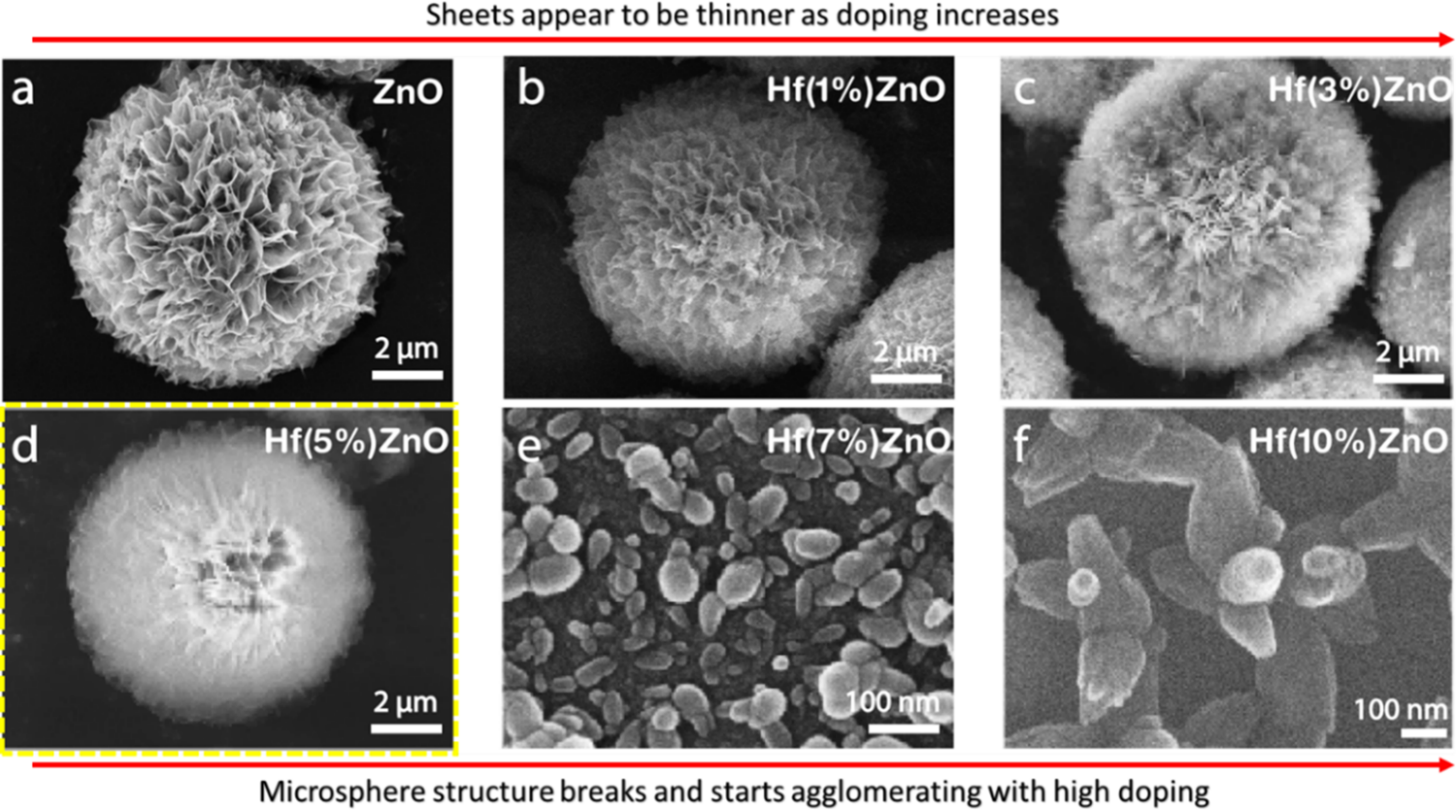
FE-SEM images of (a) ZnO and (b–f) 1, 3, 5, 7, 10% Hf-ZnO microstructures.

The FE-SEM images of the as-synthesized pristine and Hf-ZnO microstructures are shown in Figure 3. The pure ZnO and 1, 3, and 5% Hf-ZnO (Figure 3a–d) samples are thin nanosheet-assembled microspheres, forming a unique flower morphology, measuring ca. 6–7 μm in diameter.

The detailed growth mechanism of porous nanosheets assembled ZnO microsphere from the precursor is well documented in ref (22), as shown in the below equations
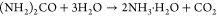
2
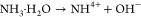
3
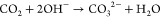
4
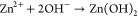
5
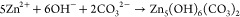
6
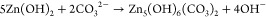
7
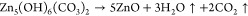
8It was illuminated that calcination of zinc hydroxide carbonate precursor in different ambient environments, air, or vacuum, brought profound alterations to the morphology and oxygen vacancy content of the ZnO nanostructure, accompanied by the evaporation of H_2_O and CO_2_, bringing in the porous nature of the ZnO morphology. It was evident that calcination in vacuum resulted in higher porosity, smaller particle size, and hence better sensing behavior. Here, further modification of vacuum-annealed ZnO microspheres with hafnium doping showed modulation of the morphology to a whole new extent.

It was observed that the morphology of the pristine ZnO (Figure 3a) was consistent until 5% of Hf doping to ZnO. Although the flower morphology was maintained, the nanosheets appeared to be thinner with increasing doping concentration. The sheets appeared to be the thinnest for the 5% Hf-ZnO microsphere, which can prove to be a very promising material toward NO_X_ gas sensing owing to its larger surface area, defects, and porosity of the sheets (Figure 3d). This is because until 5% doping, the Hf^4+^ dopants substituted well into the ZnO matrix without the formation of any secondary oxides and without deforming the lattice structure and only resulted in a decrease in particle size due to charge mismatch (shown via TEM). The morphology of the Hf-ZnO was transformed from microspheres into microparticles and then into caltrop-shaped particles with 7 and 10% Hf doping, respectively (Figure 3e,f). Further doping of ZnO with 7% Hf distorted the morphology, as the sheets detached from the spheres and agglomerated as larger and slightly oval particles with a diameter of ∼50 nm, as shown in Figure 3e. These particles with a high surface energy generally tend to agglomerate, and thus further additional doping with 10% Hf resulted in the agglomeration of the nanoparticles, mostly due to the growth mechanism involving Ostwald’s ripening and high surface energy, thereby resulting in adjuration of the particles to grow into caltrop-like microstructures. Studies^28,29^ showed similar morphology alteration owing to the incorporation of dopants. Thus, tuning doping concentration can result in morphology alteration.

**Figure 4:**
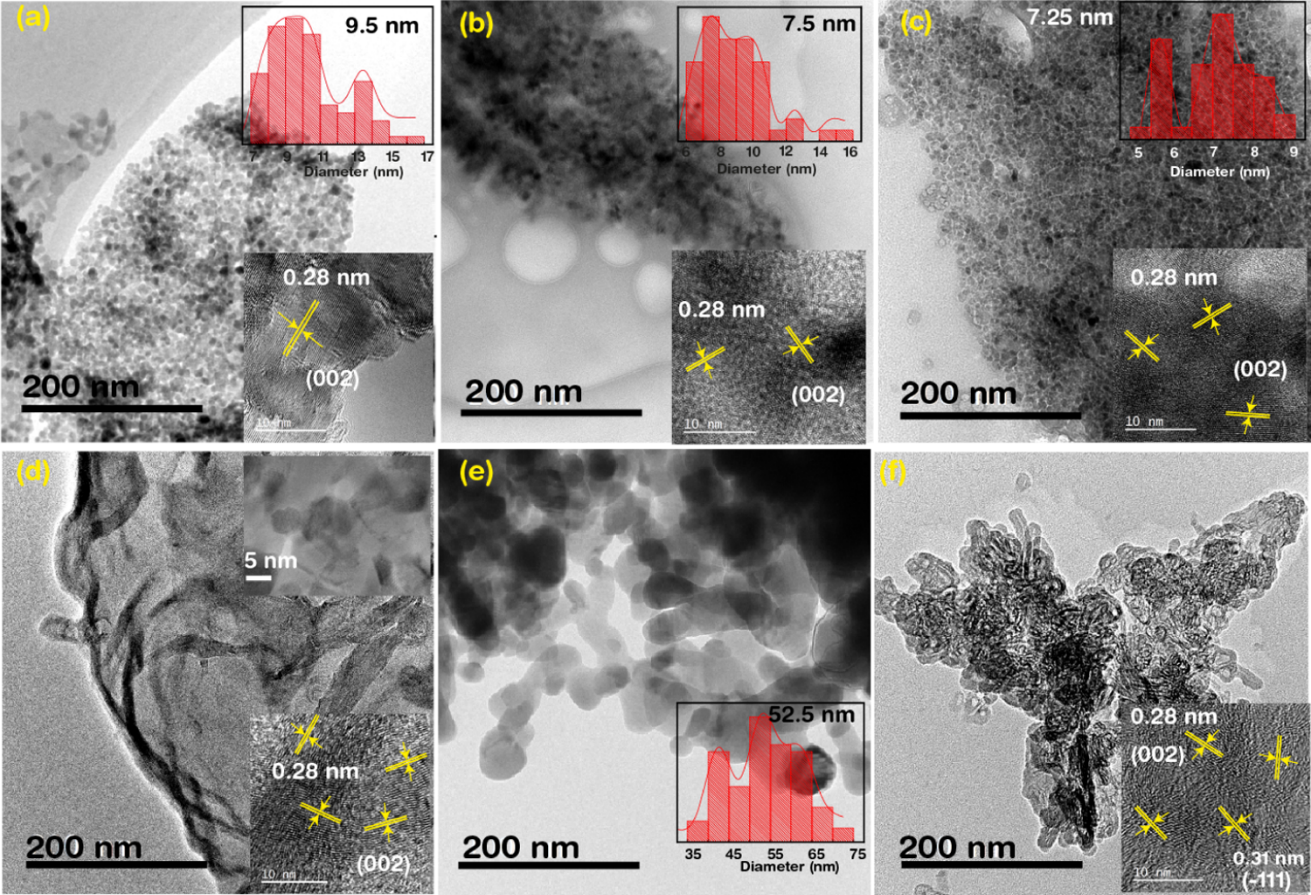
TEM analyses of (a) ZnO and (b–f) 1, 3, 5, 7, and 10% Hf-ZnO microstructures. The insets show the statistical size distributions of the samples (red bar graph) and HRTEM images displaying the lattice fringes and corresponding planes of the samples.

Further analyses utilizing TEM permitted us to shed light on the comprehensive characteristics of the samples, as shown in Figure 4. For the pristine and 1, 3% Hf-ZnO samples, the nanosheets composed of small nanoparticles carelessly interlaced, forming a large number of pores. The main difference between these samples was the nanoparticle size. Figure 4 represents the statistical size distribution of the samples (inset) by histogram representation. The average particle sizes of the pristine ZnO were 9.5 nm, which was 2 nm bigger than 1 and 3% Hf-ZnO, which had particle sizes of 7.5 and 7.25 nm, respectively. The thin sheets of 5% Hf-ZnO sample seemed to be highly porous with fringes along the edges, and particles of ∼5 nm were observed. The 7% Hf-ZnO sample is composed of quite large, interconnected particles with an average particle size distribution of 52.5 nm, much larger than the pristine ones. Finally, the 10% Hf-ZnO sample is composed of very fine sheets that were closely wound within themselves, acquiring a caltrop-like structure. The inset shows high-resolution images of all of the samples, displaying lattice fringes (*d*_spacing_ = 0.28 and 0.315 nm), relating to the (002) and (1̅11) planes of the wurtzite phase of ZnO and the cubic phase of HfO_2_ (Figure 4f), thereby confirming the segregation of HfO_2_.^30^^30^ Mean particle size reduction of the Hf-ZnO microstructures than that of the pristine and the formation of a highly porous morphology at 5% Hf-ZnO contributed toward improved gas sensing response. High porosity and small particle size control the diffusion of gases and surface reactions, as the reduction in particle size increases the active surface area and porosity. From the BET data, the specific surface areas of ZnO and 1, 3, 5, 7, and, 10% Hf-ZnO samples were estimated to be 65.3, 67.8, 69.6, 82.9, 8.9, and 19.02 m^2^/g, respectively. As anticipated, the results exposed that the samples (5% Hf-ZnO) with smaller particle sizes showed the largest specific surface areas.

### 3.2 Pure and Doped Hf-ZnO Porous Microstructures: Defect Analysis (Oxygen Vacancies)

**Figure 5:**
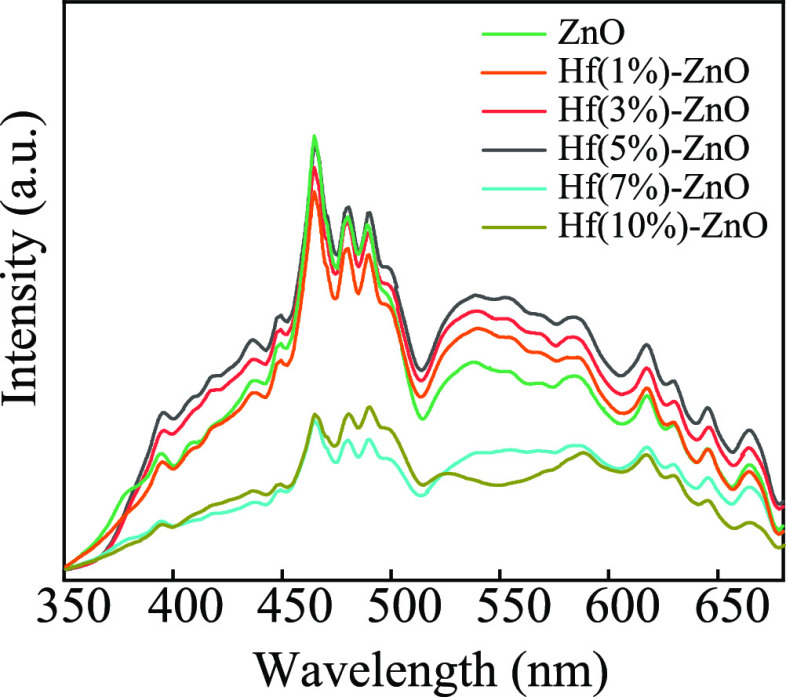
PL spectra of ZnO and 1, 3, 5, 7, 10% Hf-ZnO microstructures.

The PL spectra obtained at room temperature for pristine and Hf-ZnO samples are exhibited in Figure 5 (λ_ex_ = 325 nm). A broad green emission (deep level emission, DLE) was detected at 530 nm, which is mostly associated with the defects related to oxygen vacancy (V_O_) at the surface of samples.^31,32^ No alteration in the position of the peaks and intensity enhancement of the DLE peak with increased doping concentration from 1 to 5 wt % with further reduction in peak intensity with a higher doping concentration (7–10 wt %) confirmed the variation in defect level within the synthesized materials. The reduction in DLE peak intensity was observed due to enhanced scattering of photons by doping-induced defects.^31^ Furthermore, doping resulted in the formation of various defect levels which behave as trapping centers, thereby enhancing the recombination sites. Consequently, the intensity of the green emission band was expected to increase for the sample with a 1–5% Hf dopant, indicating a higher concentration of oxygen vacancies. Hf ions substitute Zn without phase deformation of the host ZnO up to a 5% doping concentration. Hence, enhanced gas sensing properties were observed for 5% Hf-ZnO NPs. Further doping (7–10% Hf) resulted in the collapse of the flower morphology, accompanied by decrease in oxygen vacancy concentration leading to a decrease in DLE peak intensity.^29,31^

**Figure 6:**
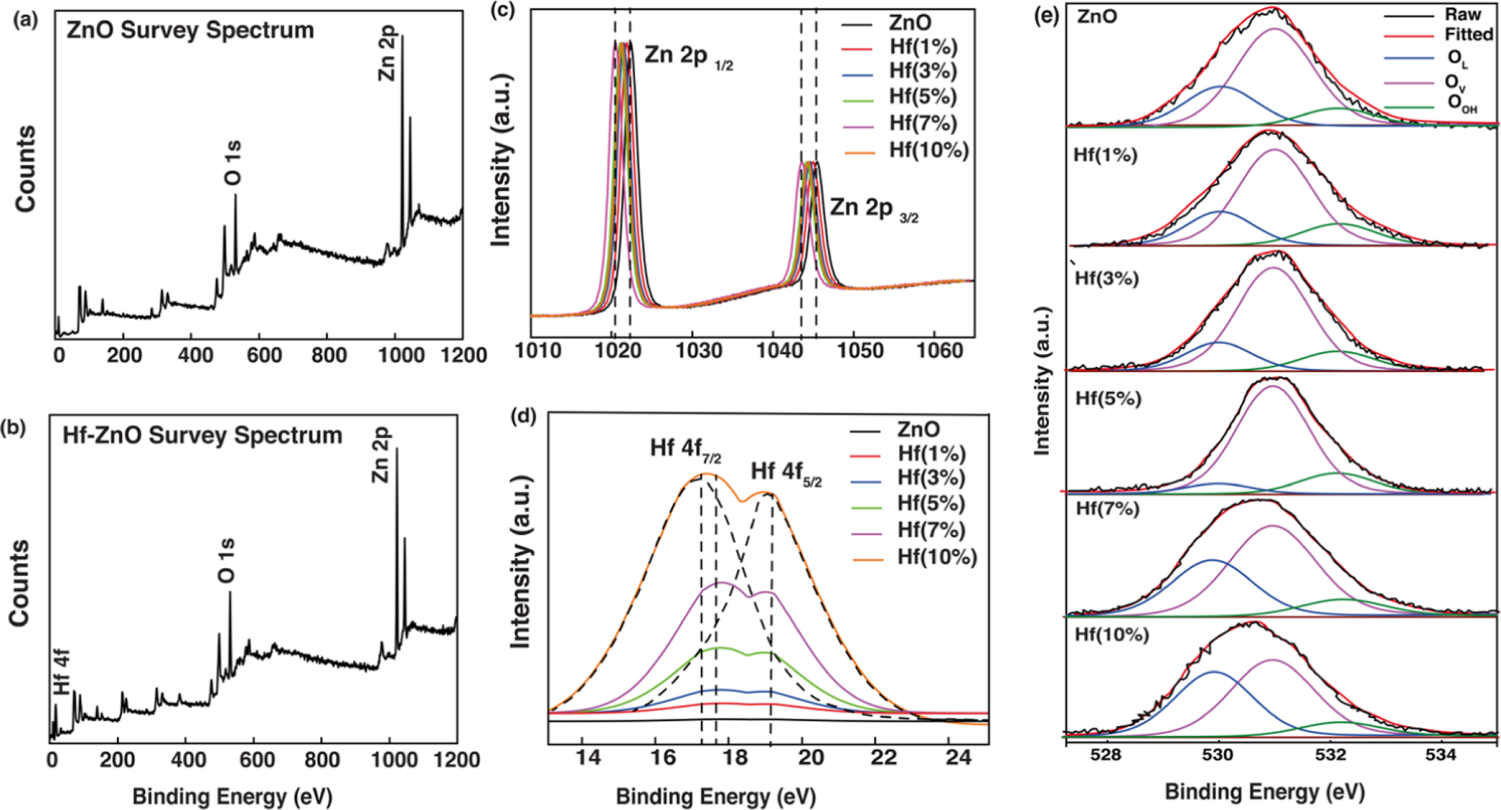
XPS spectra of (a) ZnO and (b) 7% Hf-ZnO samples. Zn 2p spectra (c), deconvoluted Hf 4f spectra (d), and deconvoluted O 1s spectra (e) of all pure and doped ZnO samples.

The XPS spectra of pristine and Hf-ZnO samples are demonstrated in Figure 6. The Zn 2p spectra of all of the samples are shown in Figure 6c. A slight shift of Zn 2p peaks was observed with increasing doping concentration, owing to lattice distortion.^14^ Studies already showed a shift in the XPS spectra peaks of doped ZnO samples.^33,34^ For instance, a shift of ∼1 eV was observed for the peaks in the XPS spectrum of 2% Co-doped ZnO sample.^35,36^ Other reasons associated with this shift have been ascribed to charge transfer, particle size, and lattice strain in nanoparticles.^35^ A clear development of the Hf 4f peaks at binding energies (BEs) 17 and 19 eV associated with the Hf^4+^ oxidation state is shown in Figure 6d, confirming the presence of Hf in the ZnO lattice with increasing Hf doping concentration, initially by substitution of Hf ions and then by the formation of HfO_2_ phases at a higher doping concentration. The differences in the O 1s region of the XPS spectra of various samples are shown in Figure 6e. According to the literature, the presence of dominant peaks at 530.05 eV (O_L_), ∼531.06–531.58 eV (O_V_), and 532 eV is attributable to the presence of oxygen in the ZnO lattice, surface defects (oxygen vacancies), and presence of oxygen ions on the surface of the ZnO, corresponding to adsorbed O_2_ or H_2_O referred to as O_OH_.^32,37,38^ The total areal percentage of each peak was calculated using the Gaussian fitting, which clearly hinted toward an increase in V_O_ concentration with an increase in doping from 1 to 5% and a decrease with further doping (7–10%). Table 2 displays the fraction of O_V_/(O_L_ + O_V_ + O_OH_) in all of the samples, calculated from the O 1s spectra, where 5% Hf-ZnO shows the presence of the highest oxygen vacancy concentration (78.17%) than ZnO (63.16%), a crucial factor responsible for outstanding gas sensing ability. A similar trend has been observed in various reports.^32,37,38^ The formation of an oxygen vacancy introduced by Hf doping can be represented in the Kröger–Vink notation^13^
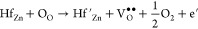
9
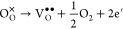
10Shannon effective ionic radii for Zn^2+^, and Hf^4+^ are 0.74 and 0.78 Å, respectively. Because of the similar ionic size, the lattice strain developed by the cation substitution is not significant. Hence, it is evident that the structure of the host lattice is stable even after the formation of atomic defects due to cation substitution. Thus, it is speculated that the V_O_ generation depends strongly on the doped Hf ions’ valence state.^39^ The incorporation of Hf (+4 valence state) in the Zn site (+2 valence state) generates charge distortion, thereby facilitating the development of oxygen vacancies in the ZnO matrix. With a higher Hf doping concentration, the Hf^4+^ ions populate the surface of ZnO, thereby reducing the valence state of Zn and alongside results in the introduction of redundant donor states, thereby enhancing the gas sensing response.^40,41^ Thus, without changing the atomic structure but increasing the surface V_O_ sites, Hf-ZnO sensors significantly improve the sensing effect. Thus, oxygen-deficient and stable transition-metal (Hf^4+^) oxide-doped stable ZnO structures can be generated as eq 2.(42)
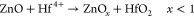
11However, a further rise in doping concentration above a critical concentration leads to the suppression of V_O_ as it induces the precipitation of a HfO_2_-rich phase, resulting in particle agglomeration.

**Table 2:** Areas of O_L_, O_V_, and O_OH_ along with Calculated Fraction of O_V_/(O_L_ + O_V_ + O_OH_) in All of the Samples from the O 1s Spectra

sample	area O_L_	area O_v_	area O_OH_	ratio % = O_V_/(O_L_ + O_V_ + O_OH_)
ZnO	7930	18 670	2960	63.16
1% Hf-ZnO	6570	18 680	3860	64.17
3% Hf-ZnO	5450	20 140	3860	68.39
5% Hf-ZnO	1950	20 800	3860	78.17
7% Hf-ZnO	10 920	17 720	3430	55.25
10% Hf-ZnO	13 650	15 940	3400	48.32

### 3.3 Gas Sensing Properties of Pure and Hf-ZnO Samples to NO_X_ and Other Gases

**Figure 7:**
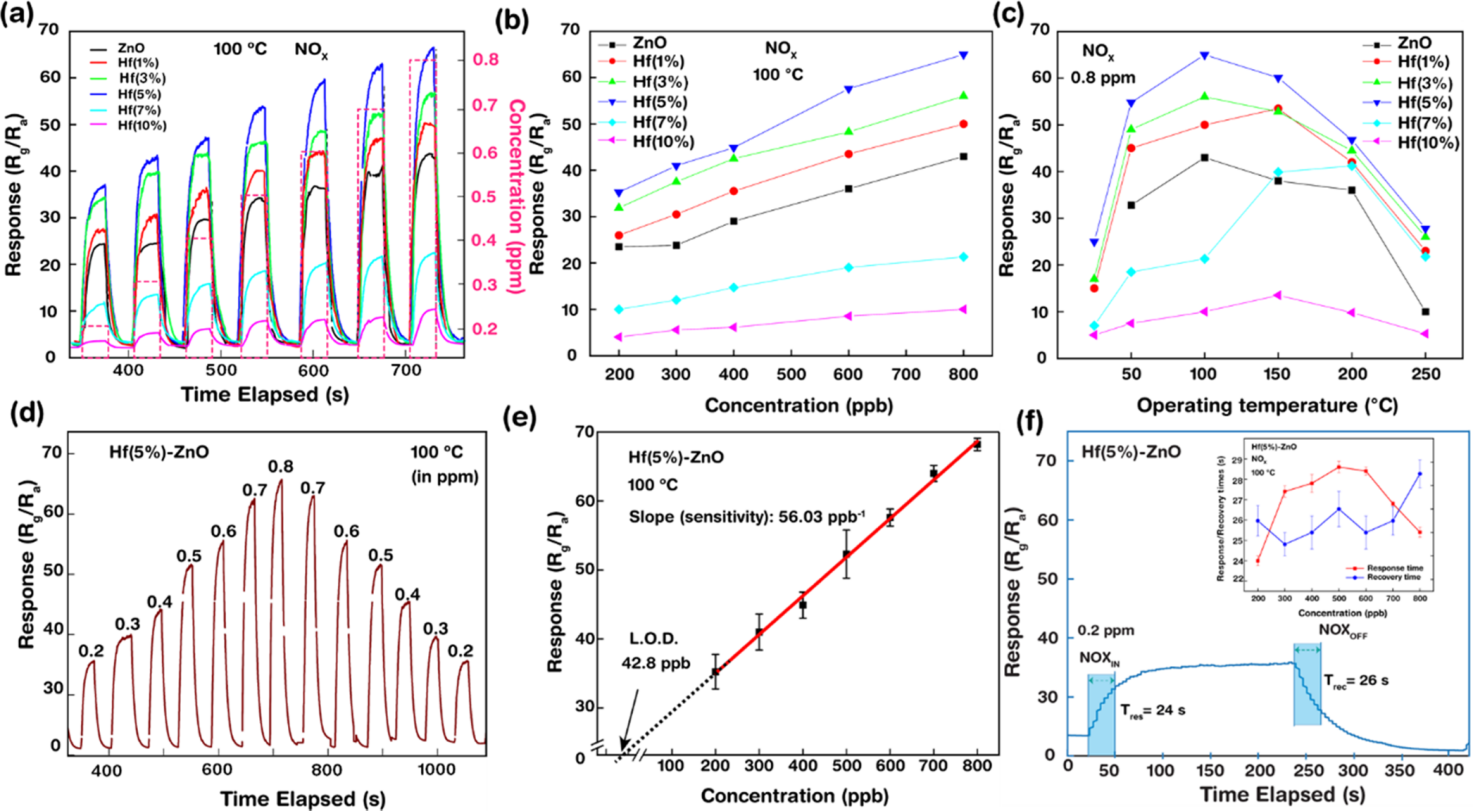
Gas sensing behavior of pure and Hf-ZnO gas sensors: (a, b) dynamic gas sensing responses and relation curve of all gas sensors to different concentrations (0.1–0.74 ppm) of NO_X_ gas at 100 °C. (c) Relationship curve of all samples to 0.8 ppm of NO_X_ gas at different operating temperatures, (d) dynamic behavior of 5% Hf-ZnO gas sensor to different concentrations (0.1–0.74 ppm) of NO_X_ gas at 100 °C for two cycles, and (e) limit of detection (LOD) and sensitivity of the 5% Hf-ZnO gas sensor derived from the extrapolation of the linear curve and slope of the line, respectively. (f) Response and recovery times of 5% Hf-ZnO gas sensor to all concentrations of NO_X_ gas at 100 °C.

The gas sensor responses of pristine and Hf-ZnO samples to various concentrations of NO_X_ at different operating temperatures are presented in Figure 7. The active gas sensing response and the linear relationship of all of the pure and Hf-ZnO gas sensors to different concentrations of NO_X_ (0.2, 0.3, 0.4, 0.6, and 0.8 ppm) at 100 °C are shown in Figure 7a,b. The gas response values linearly increased with increasing NO_X_ concentration, and the response of the 5% Hf-ZnO gas sensor was the highest at all concentrations of gas compared with any other sample. The responses of 5% Hf-ZnO toward 0.2, 0.3, 0.4, 0.6, and 0.8 ppm of NO_X_ at 100 °C were 35, 40, 42, 58, and 67, respectively, which were significantly higher than the sensing responses of pristine ZnO (21, 22, 29, 32, and 40). The responses of the 1, 3, and 5% Hf-ZnO gas sensors were higher than the pure ZnO gas sensors, whereas the responses of the 7 and 10% Hf-ZnO samples drastically dropped below the response of the pure ZnO gas sensor. This was attributed to the higher surface area, porosity, and presence of oxygen vacancies in 1–5% Hf-ZnO. The responses of all of the samples were observed at various operating temperatures (25, 50, 100, 150, 200, 250 °C). Among all of the samples, the 5% Hf-ZnO gas sensor reached the maximum response (*S* = 67) at 100 °C (Figure 7c). Therefore, all further measurements were performed at 100 °C as the response decreases drastically beyond 100 °C. For the responses recorded at lower temperatures (<100 °C), the rate of reaction of NO_X_ gas with the sensor surface is reduced attributed to inadequate thermal energy. However, at higher operating temperatures (⩾150 °C), desorption of gas molecules is relatively faster than adsorption, thereby also decreasing the gas sensing response. Thus, at an optimized temperature of 100 °C, both the adsorption and desorption processes are equivalent, exhibiting maximum gas sensing response. It is also worth noticing that room-temperature detection is possible with the 5% Hf-ZnO gas sensor. The reproducibility of the 5% Hf-ZnO gas sensor was explored by measuring the sensor response for repeated cycles over different concentrations. As shown in Figure 7d, a similar response was observed for all of the measurements. The lowest point of detection or limit of detection (LOD) of the 5% Hf-ZnO was evaluated by plotting the linear curve of the gas sensing response versus concentration of the gas and then extrapolating the straight-line portion to the concentration axis at response = 0 (Figure 7e). The LOD was determined to be 22.8 ppb. Furthermore, the sensitivity of the sensor (56.03 ppb^–1^) was estimated from the slope (response vs. concentration). The sensor’s sensitivity was significantly improved by doping it with 5% Hf. The response and recovery times as shown in Figure 7f are the two crucial features responsible for sensor’s performance evaluation. The times required by a sensor to reach either 90% of its maximum point when exposed to analyte gas or 10% toward the baseline after stopping the gas flow are denoted as response and recovery times. The response/recovery times of the 5% Hf-ZnO gas sensor were 24/26 s, which were significantly faster than the pristine ZnO gas sensor (90/100 s). This showed that the 5% of Hf-ZnO gas sensor was more sensitive to NO_X_ than the pure ZnO gas sensors.

**Figure 8:**
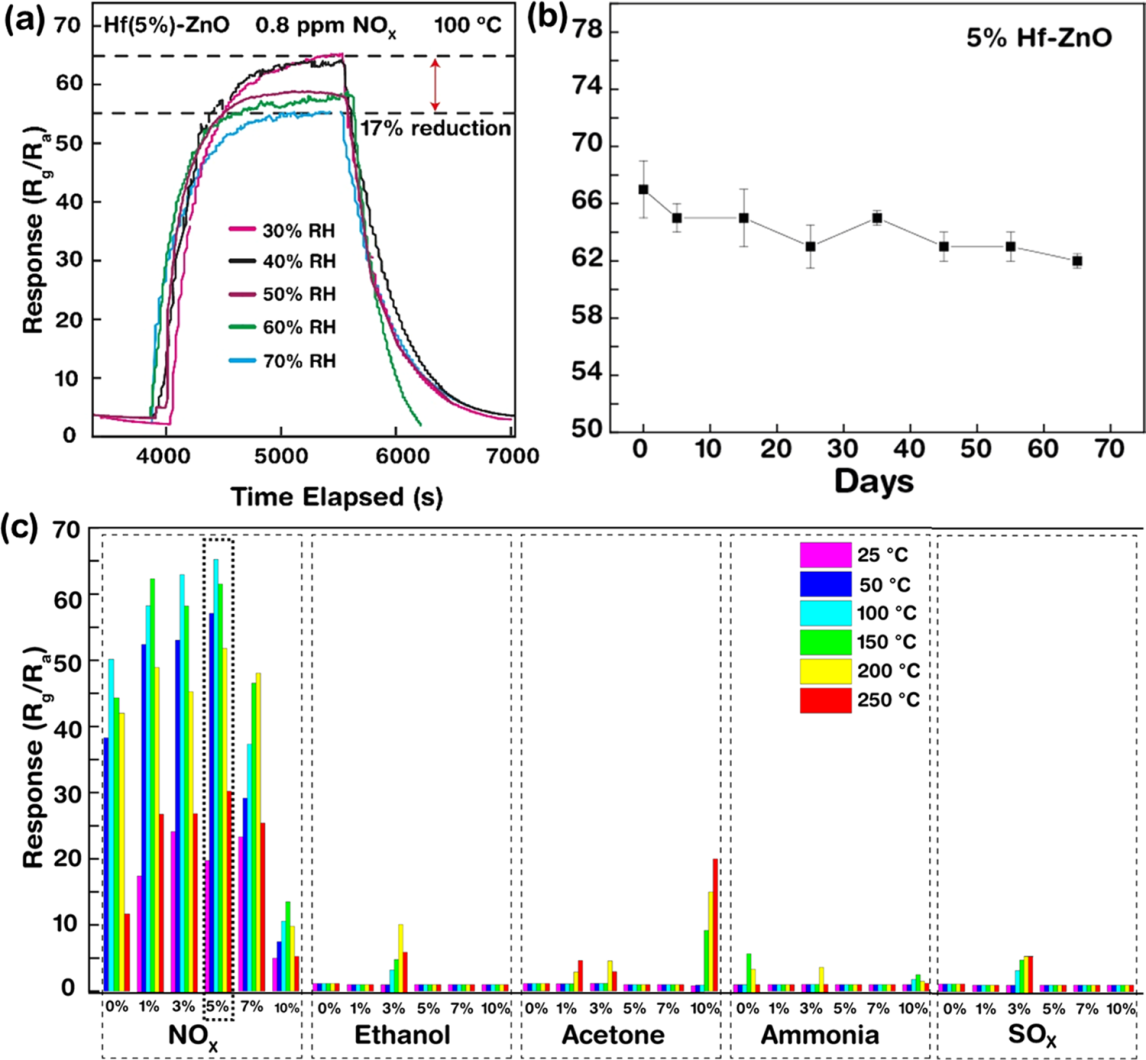
(a) Gas sensing measurements of 5% Hf-ZnO towards 0.8 ppm of NO_X_ at 100 °C under different humidity conditions: 30, 40, 50, 60, and 70% RH; (b) stability of the gas sensor over 65 days; and (c) selectivity of pure and Hf-ZnO gas sensors to 0.8 ppm of NO_X_, 4.5 ppm of ethanol, acetone, ammonia, and SO_X_ gases at different operating temperatures (50, 100, 150, 200, and 250 °C).

Further gas sensing measurements of 5% Hf-ZnO toward 0.8 ppm of NO_X_ were conducted in a humidity-controlled chamber under different RH, ranging from 30 to 70% RH, at 100 °C, to check the long-term stability of the 5% Hf-ZnO sensor (Figure 8a). The sensor response remains constant till 40% RH with slight decreases in response (58) at 50% RH and 56 and 55 at 60 and 70% RH. A total of 17% reduction in response is observed under high humidity conditions. This phenomenon is observed due to the presence of water molecules in the air, which gets adsorbed onto the surface of Hf-ZnO, thereby reducing the number of available active sites for the gas interaction. The response is recovered after keeping the sensor in a desiccator for 5 h, hinting toward the reusability of the sensor. Additionally, the long-term stability of the sensor was also verified, as the sensor showed very stable behavior over a period of 65 days (∼2 months), where the sensor was kept in the desiccator and taken out at regular intervals for the test. Finally, gas selectivity, also an important parameter for evaluating gas sensor performance, was investigated for all of the samples toward NO_X_, ethanol, acetone, and ammonia. The results (Figure 8c) indicated that all of the samples, especially the 5% Hf-ZnO gas sensor, had very good selectivity to NO_X_, making it feasible to distinguish NO_X_ gas as a specific target gas amidst the mixture.

Thus, a 5% Hf-ZnO gas sensor showed fast and the highest gas response with high stability and selectivity toward NO_X_ gas. Therefore, modulating the Hf doping concentration altered not only the morphological and microstructural properties but also the gas sensing behavior. It is evident from the results that efficient charge transfer between the dopant ion and the host lattice due to charge mismatch (Hf^4+^ and Zn^2+^), high porous morphology (grain size refinement), surface area increments, and strong basic nature of Hf, leading to Lewis acid interaction, which are the main contributing factors for promoting oxygen vacancies formation, result in an increase in the number of adsorption sites. This in turn enhances the gas sensing behavior of 5% Hf-ZnO over the pristine sample.

### 3.4 Gas Sensing Mechanism with Density Functional Theory (DFT) Validation

Thus, here, we fabricated highly sensitive and selective NO_X_ gas sensor with Hf-doped ZnO. By optimizing the hafnium doping concentration, we modulated the morphology and oxygen vacancy concentration, which enhanced the performance of the sensor almost 2-fold (67 at 100 °C toward 0.8 ppm of NO_X_) with 5% Hf. It is quite noteworthy to observe that both sensor performance and oxygen vacancy concentration depend on the doping percentage of hafnium. Both response and defects increased with an increase in doping until 5%, beyond which they started declining (10%). Thus, it is well established that oxygen vacancies induced due to doping with Hf played a pivotal role in increasing NO_X_ sensing.

**Figure 9:**
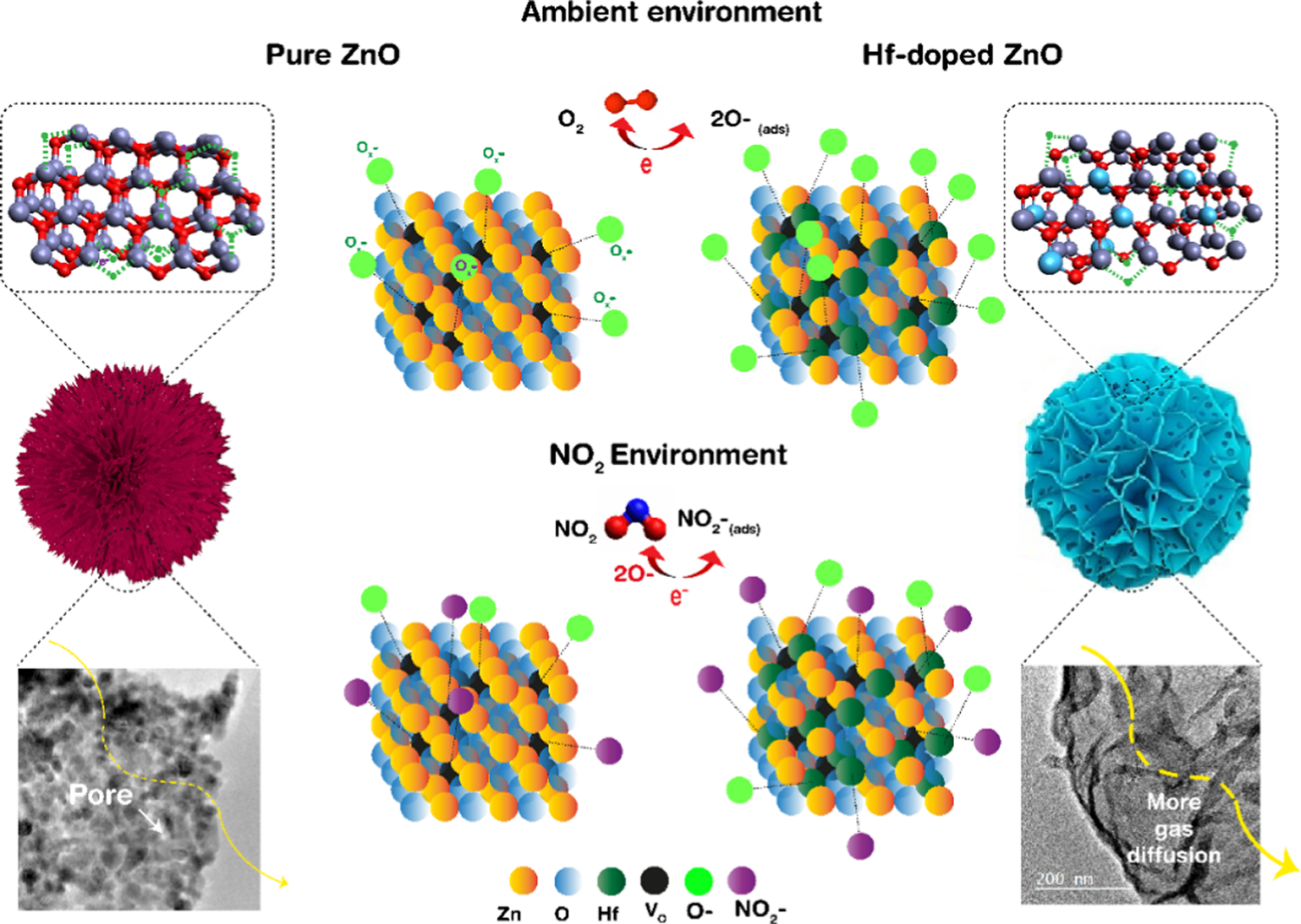
Schematic diagram representing the sensing mechanism of pure and 5% Hf-ZnO microsphere to air and NO_X_ gas environments.

In general, under ambient exposure and depending on the ambient temperature, oxygen molecules, which are adsorbed on the surface of the sensing material, possess different charge states (O_2_^–^, O^–^, and O^2–^) due to ionization arising due to the extraction of electrons from the conduction band and hence trapping them onto the surface, thereby resulting in resistance change of the gas sensor.^43^
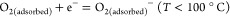
12This oxygen-adsorbed surface of ZnO is exposed to the NO_X_ gas, which in turn extracts more electrons by reacting with the adsorbed oxygen species (NO_X_ has an elevated electron affinity of ∼2.28 eV > 0.43 eV than that of oxygen),^44−46^ thereby causing a sharp rise in resistance and increasing the depletion layer.
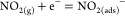
13This interaction rises between NO_X_ molecules, and the adsorbed oxygen species increases drastically, leading to higher resistance change, due to the provision of more adsorption sites owing to an increase in a number of oxygen vacancies. Oxygen vacancies or defect-enriched sites are preferential adsorption sites for NO_X_ molecules (41.4 kcal/mol) than the nondefective sites (13.8 kcal/mol), which are higher in concentration in Hf-doped ZnO than in pristine ZnO. From Figure 9, the relation between high gas response at a low temperature and a high oxygen vacancy concentration in 5% Hf-ZnO can also be observed. In addition, the high basicity of the sensing material, due to the addition of hafnium increased the interaction with the NO_X_ molecule, being acidic in nature.

Apart from XPS, density functional theory (DFT) was also employed to investigate the impact of oxygen vacancies on the NO_X_ sensing caused by the Hf doping.

**Figure 10:**
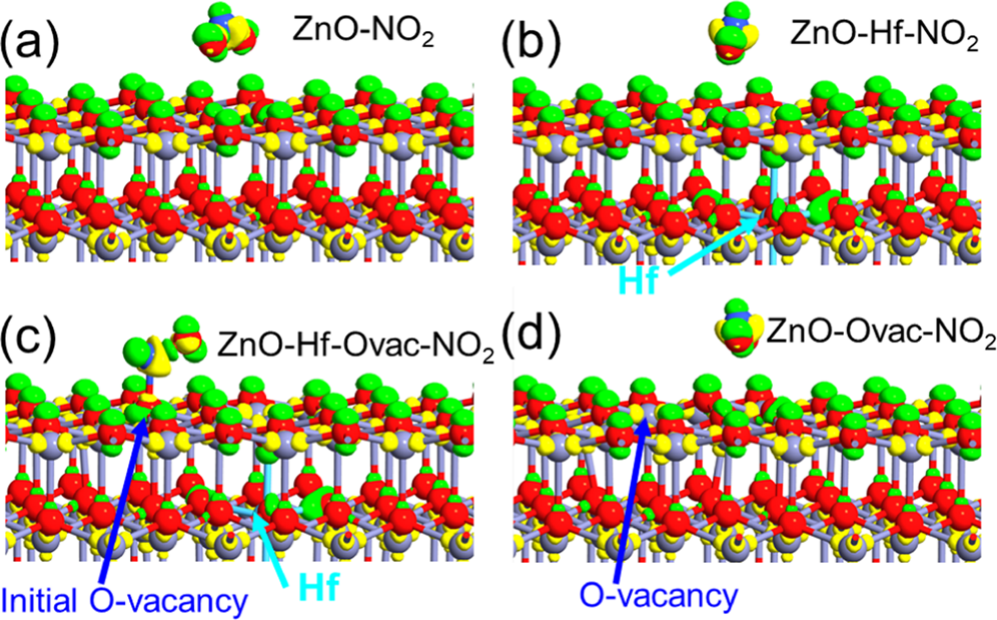
NO_2_-supercell electron density difference superimposed on the geometrically optimized atomic configuration of (a) ZnO without any defect, (b) ZnO with one Hf atom substitutional at the Zn position, (c) ZnO with one Hf atom substitutional at the Zn position, and an oxygen vacancy at the top surface, (d) ZnO with one oxygen atom vacancy in the top surface. Iso value: 0.15 e/Å.^24^ Green color indicates the electron-rich area, and yellow color depicts the electron depletion region. Red, light violet, dark blue, and light blue balls denote O, Zn, N, and Hf atoms, respectively.

The (101̅0) crystal face of the hexagonal ZnO is cleaved, and the supercell slap with 360 atoms is created for all of the simulations (Supporting Movies show details of the atomic structure). The resultant dimensions of the slap supercell were 19.497 Å × 16.8849 Å × 40 Å. Four different types of supercells were constructed: (1) ZnO without any defect (ZnO-NO_2_), (2) ZnO with one Hf atom substitution at the Zn position (ZnO-Hf-NO_2_), (3) ZnO-Hf-NO_2_ with an oxygen vacancy at the top surface (ZnO-Hf-O_vac_-NO_2_), and (4) ZnO with one oxygen atom vacancy on the top surface (ZnO-O_vac_-NO_2_). First, these atomic configurations were geometrically optimized by relaxing the atomic structures until the remaining residual force was smaller than 0.05 eV/Å. During these optimizations, the bottom two layers were fixed in position. In all of these atomic structures, initially, the NO_2_ molecule was placed in the same position and orientation above the ZnO slap. These atomic configurations are shown in Figure 10 and Supporting Movies. In the configuration of ZnO without any defect, the oxygen atoms of the NO_2_ molecule moved downward during the structure optimization and NO_2_ made physisorption bonding with the topmost layer of ZnO. In the case of ZnO-Hf-NO_2_, the NO_2_ molecule bent downward and made a physisorption bonding with the topmost layer of ZnO. However, in the case of ZnO-Hf-O_vac_-NO_2_, the NO_2_ molecule moved toward the oxygen vacancy and made a chemical bond with the ZnO layer. To confirm the role of the Hf atom in this chemisorption, ZnO with only an oxygen vacancy (i.e., ZnO-O_vac_-NO_2_) was considered. During this structure optimization, the NO_2_ molecule did not move toward the oxygen vacancy, instead, it made physisorption-type bonding with the topmost layer of ZnO.

**Figure 11:**
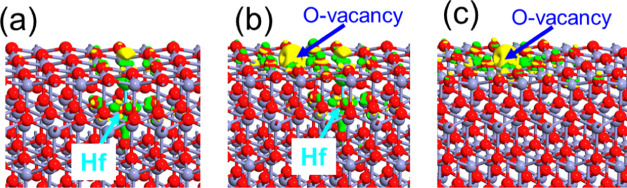
Electron density difference of the supercells (a) ZnO-Hf, (b) ZnO-Hf-O_vac_, and (c) ZnO-O_vac_ with respect to the ZnO supercell, respectively.

To understand the charge transfer between NO_2_ molecule and ZnO, Mulliken population analysis was carried out. In the case of ZnO-NO_2_, ZnO-Hf-NO_2_, and ZnO-O_vac_-NO_2_, a charge of 0.259, 0.113, and 0.217 e, respectively, was transferred from the NO_2_ molecule to ZnO. As the topmost layer, oxygen atoms of ZnO lack Zn electron contribution and they take electrons from the NO_2_ molecule and the atoms around the oxygen vacancy position rearrange. In the structure with one Hf substitutional with an oxygen vacancy, the NO_2_ molecule received 0.288 e from the ZnO-Hf-O_vac_ supercell. This was attributed to the availability of electrons from the Hf atom and the presence of an oxygen vacancy. The electron density difference of the supercells ZnO-Hf, ZnO-Hf-O_vac_, and ZnO-O_vac_ with respect to the ZnO supercell is plotted in Figure 11. The case of a supercell with substitutional Hf atom depicts the presence of the extra electron density around the Hf atom (Figure 11a). Electrons are depleted around the oxygen vacancy position of the ZnO-O_vac_ supercell (Figure 11b). Especially, when the substitutional Hf atom and the oxygen vacancy are present, then the electron density is depleted at the oxygen vacancy position and extra electron density exists around the Hf atom. This combination attracts the NO_2_ molecule toward the oxygen vacancy and then leads to stronger bonding. Therefore, the presence of an oxygen vacancy induced by Hf atom doping leads to chemisorption of the NO_2_ molecule.

The binding energy of the NO_2_ molecule on the supercells of different ZnO is calculated as *E*_Bind_ = *E*_(ZnO-X/Nb-NO_2_)_ – (*E*_ZnO-X_ + *E*_NO_2__), in which *E*_(ZnO-X/Nb-NO_2_)_ is the total energy of the NO_2_ molecule adsorbed on the ZnO-NO_2_, ZnO-Hf-NO_2_, ZnO-Hf-O_vac_-NO_2_, and ZnO-O_vac_-NO_2_ supercells; *E*_ZnO-X_ is the total energy of the ZnO, ZnO-Hf, ZnO-Hf-O_vac_, and ZnO-O_vac_ supercells; and *E*_NO_2__ is the total energy of NO_2_ molecule. The calculated NO_2_ molecule binding energies were 1.31722, 1.08681, 2.87061, and 1.11938 eV for ZnO-NO_2_, ZnO-Hf-NO_2_, ZnO-Hf-O_vac_-NO_2_, and ZnO-O_vac_-NO_2_ atomic structures, respectively. These binding energies indicate weaker bonding of the NO_2_ molecule with the ZnO, ZnO-Hf, and ZnO-O_vac_ materials and stronger bonding of the NO_2_ molecule with the ZnO-Hf-O_vac_ material. These results explain the high sensitivity and excellent selectivity observed in the samples with Hf doping (5 wt %) ZnO oxygen vacancies. On the other hand, the binding energy of the oxygen atom to the NO_2_ molecule was calculated to be 6.3216 eV, which was much higher than the binding energy of the NO_2_ molecule to the ZnO-Hf-O_vac_ material. The atomic distances of the bonded oxygen atom of the NO_2_ molecule to ZnO were relatively longer than the oxygen atom distance in ZnO (see Supporting Information Figure S1). Because of this higher binding energy of the oxygen atom to the NO_2_ molecule, longer bonding length, high-temperature sensing measurements, and fast response and recovery might be possible.

## 4 Conclusions

Pristine and Hf-ZnO gas sensors were prepared using hydrothermal synthesis. Doping with hafnium played a significant role in modifying the morphology of the porous nanosheet-assembled ZnO flowerlike nanostructure and enhancing the NO_X_ gas sensitivity. Detailed characterization showed successful substitutional doping up to a 5%, which significantly increased the amount of V_O_ (evident from PL and XPS), where XPS showed the highest concentration of V_O_ in 5% Hf-ZnO (78%), then ZnO (63%), and further a particle size reduction was observed (as confirmed by XRD and TEM: 5 nm for 5% Hf-ZnO and 9.5 nm for pristine ZnO), thereby enhancing the specific surface area (BET: 82.9 m^2^/g for 5% Hf-ZnO and 65.3 m^2^/g for pristine ZnO). DFT analysis indicated that weaker bonding (binding energy) of the NO_2_ molecule with the ZnO (1.31722), ZnO-Hf (1.08681 eV), and ZnO-O_vac_ (1.11938 eV) and stronger bonding of the NO_2_ molecule with ZnO-Hf-O_vac_ material (2.87061 eV) were the causes of the elevated sensitivity and outstanding selectivity observed in the Hf-doped (5 wt %) ZnO containing more oxygen vacancies. The superior performance of the 5% Hf-ZnO microstructure to NO_X_ gas was discovered. The sensing test fallouts directed that a suitable quantity of Hf doping (5 wt %) significantly upgraded the gas detecting properties (*S* = 68–0.8 ppm of NO_X_ gas) with elevated sensitivity (22 ppb) and outstanding selectivity, to achieve faster response and recovery times (24/26 s) than pristine ZnO (90/100 s). Hf-ZnO offered outstanding gas sensing behavior because of the presence of oxygen vacancies between host (Zn) and dopant (Hf) lattice. Hence, the gas sensing mechanism of ZnO can be optimized during the synthesis process by increasing oxygen vacancies. A strong Lewis acid–base interaction shows a significant contribution to detect NO_X_ by Hf-doped ZnO. Hafnium doping refines the grain size of ZnO and converts it to highly porous, thin nanosheets, which enhance the NO_X_ gas diffusion throughout the material, which in turn increased the number of adsorption sites and the surface area, and finally, the NO_X_ gas response is increased significantly. However, higher doping (≥7 wt %) showed deterioration of gas detecting properties of ZnO and destruction of the morphology owing to the formation of HfO_2_, which also suppressed the oxygen vacancy content and decreased the specific surface area.

## Supporting Information Available

Nearest atomic distances between the ZnO-bonded oxygen atom of NO_2_ molecule and ZnO atoms and the oxygen atom in ZnO and atomic configurations of four different types of supercells (PDF)

Figure S2 movies (ZIP)
