# Supplementary material for: Development of Morphologically engineered Flower-like Hafnium-Doped ZnO with Experimental and DFT Validation for Low-Temperature and Ultrasensitive Detection of NOX Gas
Source: Ind Eng Chem Res. 2022 Apr 22;61(17):5885–97. doi: 10.1021/acs.iecr.2c00890 (PMC9097477; doi:10.1021/acs.iecr.2c00890)
Supplement: Supplementary file 1 — ie2c00890_si_001.pdf [file ie2c00890_si_001.pdf]

Supplementary Information

Development of Morphologically Engineered Flower-like Hafnium Doped ZnO with Experimental and DFT Validation for Low-Temperature and Ultrasensitive Detection of NOx gas

Srijita Nundy,[a,f,] *, Sankar Ganesh Ramaraj[b], Manoharan Muruganathan [b], Aritra Ghosh[e] ,Asif Ali Tahir[f], Tapas Kumar Mallick[f], Joon-Shik Park[d,] *, Hoo-Jeong Lee[a, c,] *

[a]School of Advanced materials and science engineering, Sungkyunkwan University, Suwon 16419, Republic of Korea

[b]School of Materials Science, Japan Advanced Institute of Science and Technology, Nomi 923-1211, Japan

[c]SKKU Advanced Institute of Nano Technology, Sungkyunkwan University, Suwon 16419, Republic of Korea

[d] Smart Sensor Research Center, Korea Electronics Technology Institute (KETI), Seongnam 13509, Republic of Korea

[e]College of Engineering, Mathematics and Physical Sciences, Renewable energy, University of Exeter, Penryn TR10 9FE, United Kingdom

[f]Environment and Sustainability Institute, University of Exeter, Penryn TR10 9FE, United Kingdom

*Corresponding Authors

E-mails: s.nundy@exeter.ac.uk (S. Nundy) jspark@keti.re.kr (J.-S. Park), hlee@skku.edu (H.-J. Lee)

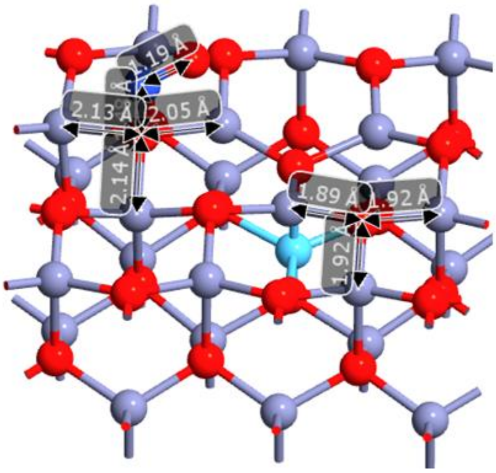

Figure S1. Nearest atomic distances between the ZnO bonded oxygen atom of NO_2 molecule and ZnO atoms and the Oxygen atom in ZnO.

ZnO-NO2.avi ZnO-Hf-NO2.avi zno-hf-Ovac-no2.avi zno-Ovac-no2.avi

Figure S2 movies. Atomic configurations of four different types of supercells: 1) ZnO without any defect (ZnO-NO_2), 2) ZnO with one Hf atom substitutional at Zn position (ZnO-Hf-NO_2), 3) ZnO-Hf-NO_2 with Oxygen vacancy at the top surface (ZnO-Hf-Ovac-NO_2), and 4) ZnO with one Oxygen atom vacancy in the top surface (ZnO-Ovac-NO_2). Initially, the NO_2 molecule is placed in the same position and orientation above the ZnO slap and interaction is observed.
